# Orbitofrontal cholesterol granuloma masquerading as frontal sinus mucoceles: report of two cases

**DOI:** 10.1186/s12886-023-02842-3

**Published:** 2023-03-13

**Authors:** Ruimiao Li, Mingyu Ren, Wenjing Wang, Ruixin Li, Lili Zhang, Limin Liu

**Affiliations:** 1 Department of Orbital Disease and Ocular Tumor, Hebei Eye Hospital, Xingtai, 054001 Hebei China; 2 People’s Hospital of Pingxiang County, Xingtai, 054001 Hebei China

**Keywords:** Orbitofrontal cholesterol granuloma, Frontal sinus mucoceles, Diagnostic imaging, Surgical therapy, Case report

## Abstract

**Background:**

Two cases of orbitofrontal cholesterol granuloma masquerading as frontal sinus mucoceles were reported to understand image findings, clinical and histopathologic features of orbitofrontal cholesterol granuloma to improve its diagnosis and treatment.

**Case presentation:**

Two East Asian patients aged 41 and 27 without personal or familial medical or trauma history presented with the common complaint of proptosis and inferomedial displacement of the eyeballs. The computed tomography (CT) of both cases showed an irregularly shaped, well-defined lesion in the left frontal bone associated with bony erosion. The lesions resulted in the bone absorption of frontal bone and orbital roof, which extended into the superior orbital space. Anterior orbitotomy through subbrow incision by drainage and curettage resulted in a curative outcome. The histopathological examination revealed inflammatory granulation tissues, fibrous capsule wall, cholesterol clefts with altered blood pigments, and calcifications, consistent with the diagnosis of cholesterol granuloma. No recurrence was observed for one year after surgery in one case and three years in the other.

**Conclusions:**

When the following features are observed: orbital CT exhibits cystic lesion with irregular bone destruction in the superolateral orbit, magnetic resonance imaging (MRI) depicts lesions are hyperintense signals on T1 weighted images (T1WI), and T2 weighted images** (**T2WI), and the contrast-enhanced imaging reveals that the most of tumor is showed a non-significant enhancement, orbitofrontal cholesterol granuloma should be considered.

## Background

Cholesterol granuloma is an osteolytic lesion with a granulomatous reaction surrounding cholesterol crystals, old hemorrhage, and a fibrous capsule [1]. It has been reported in several locations, such as the peritoneum, lungs, breast, lymph nodes, kidney, and testis [2]. Within the head and face location, it is mostly associated with bony structures, such as the mastoid antrum and air cells of temporal bone, jaw, nasal sinuses, skull base, and orbital bone [3]. Orbital cholesterol granuloma generally occurs in the frontal bone in the superolateral aspect of orbit [4]. However, the location of frontal sinus mucoceles is also similar, along with the bone invasion, which leads to misdiagnosis. The records of two patients with histologically confirmed cholesterol granuloma were reviewed. We summarized the typical clinical presentations, computed tomography (CT), and magnetic resonance imaging (MRI) results of the two patients to improve the diagnostic ability for this disease.

## Case presentation

### Case 1

**Fig. 1:**
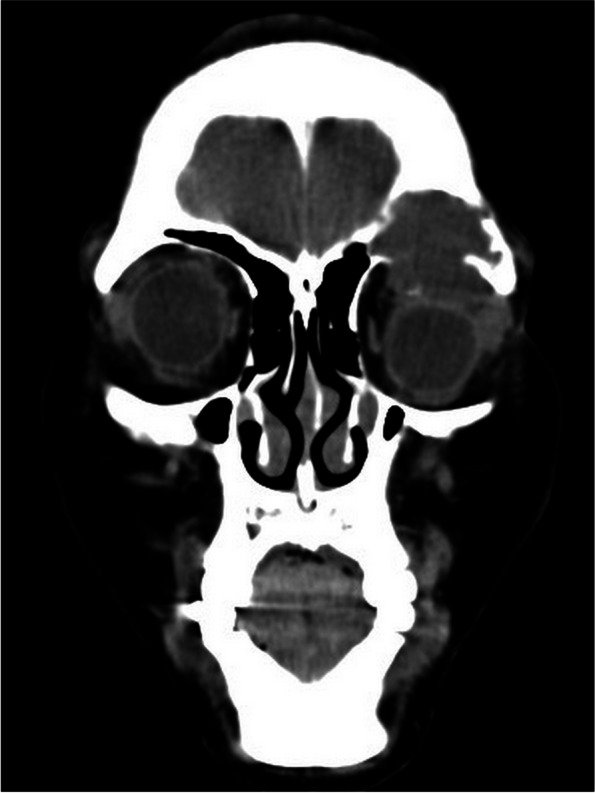
CT scan displayed a soft tissue mass in the left frontal sinus, eroding the floor of sinus, orbital roof, and extending into the superior orbital space

**Fig. 2:**
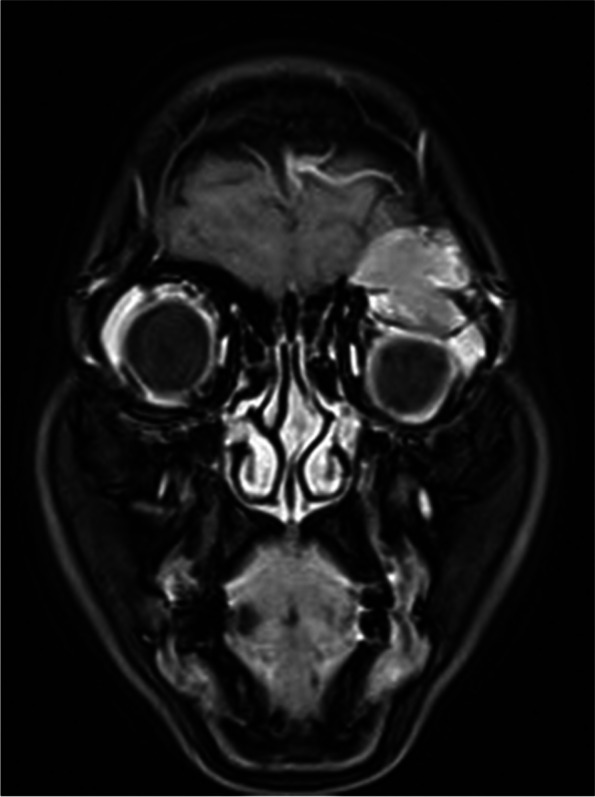
Enhanced magnetic resonance imaging showed a non-enhancing mass that presented hyperintense

**Fig. 3:**
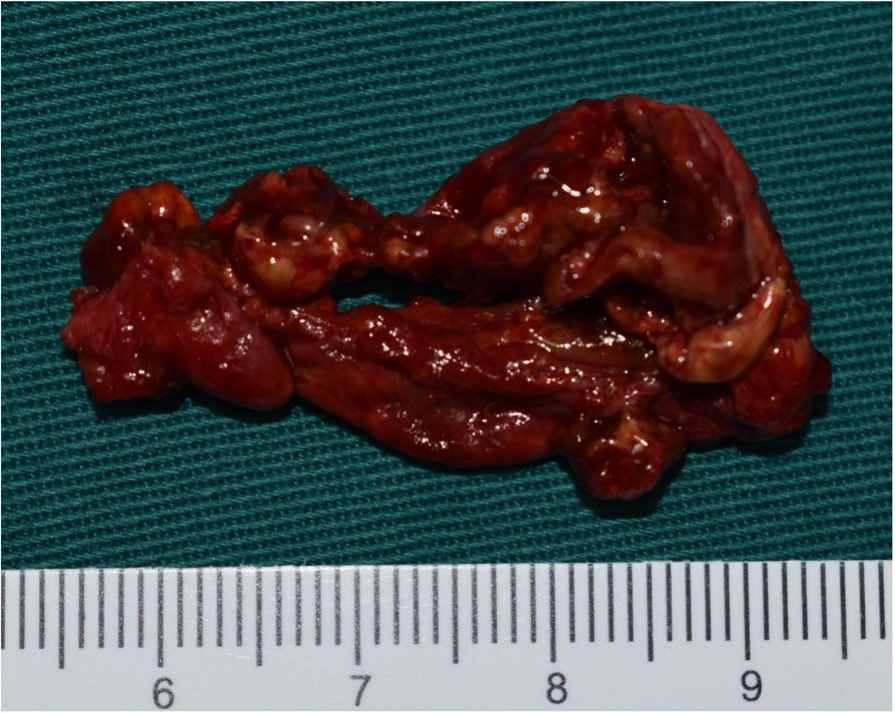
Surgical sample showed a well-circumscribed cyst

**Fig. 4:**
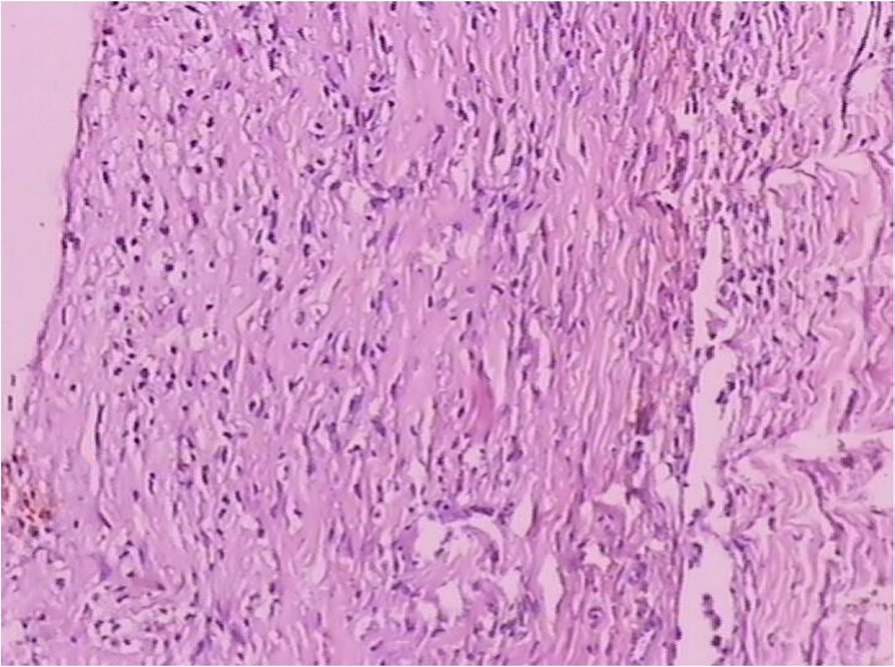
The histopathological examination of cyst contents and wall revealed inflammatory granulation tissues, fibrous capsule wall, cholesterol clefts with altered blood pigments, and calcifications, consistent with the diagnosis of cholesterol granuloma

A 41-year-old East Asian female patient with a one-year history of gradual painless proptosis in the left eye was admitted. There was no significant personal or familial medical and trauma history. Blood and urine tests were normal. She had no any disturbance in the lipid profile. The best-corrected visual acuity was 20/25 in the right eye and 20/33 in the left eye. The patient presented with painless proptosis, inwards and downward eyeball displacement, and upward restricted extraocular muscle movements. Hertel exophthalmometry readings were 16 mm in the right eye and 13 mm in the left. An irregularly shaped, well-defined soft mass was palpable in the superior aspect of left orbit. The rest of ocular examination was normal. CT scan displayed a 2.8 × 3.2 × 1.5 cm soft tissue mass in the left frontal sinus, eroding the floor of sinus, orbital roof, and extending into the superior orbital space (Fig. 1). Magnetic resonance imaging (MRI) exhibited an irregular mass, the lesions were hyperintense signals on T1 weighted images (T1WI) (Fig. 2). The lesions were isointense and hyperintense mixed signals on T2 weighted images (T2WI). Contrast-enhanced imaging demonstrated that most tumor parts reveal a non-significant enhancement. Resection was performed via an anterior orbitotomy. After incising the superolateral orbital periosteum, a cavity containing dark red liquid was accessed just inside the orbital rim. During the operation, the cystic fluid was aspirated, the cystic wall was removed (Fig. 3), and the abnormal bone was thoroughly curetted. The histopathological examination of cyst contents and wall revealed inflammatory granulation tissues, fibrous capsule wall, cholesterol clefts with altered blood pigments, and calcifications, consistent with the diagnosis of cholesterol granuloma (Fig. 4). There was no recurrence upon follow-up.

### Case 2

**Fig. 5:**
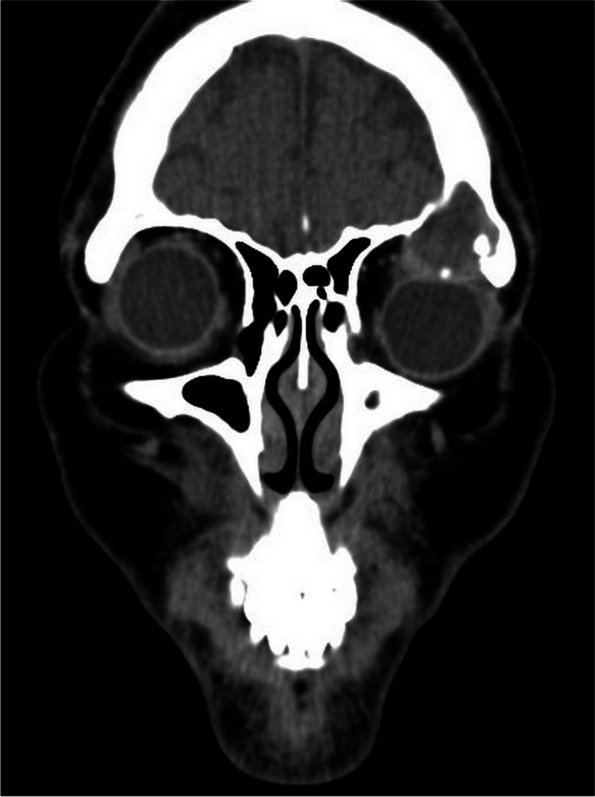
CT scan exhibited an irregularly shaped promiscuous dense occupying lesion in the left frontal bone, resulting in the bone absorption in the frontal bone and orbital roof, and penetrating down into the orbit

**Fig. 6:**
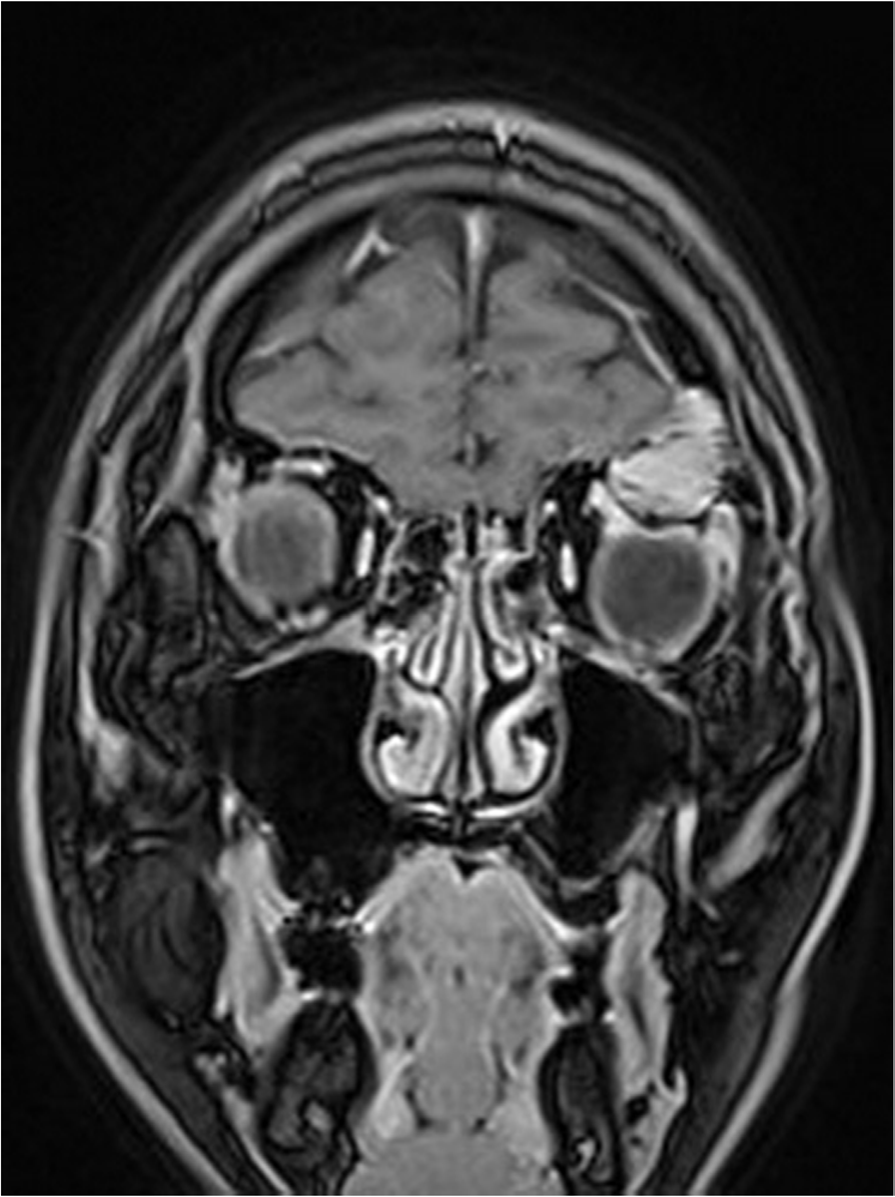
Contrast-enhanced imaging revealed that most parts of the tumor display a non-significant enhancement

**Fig. 7:**
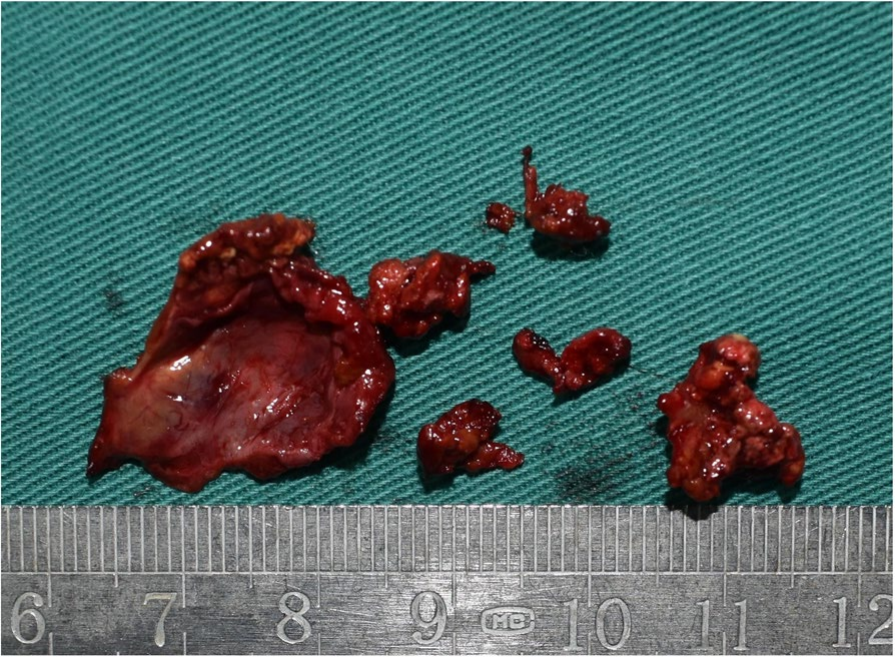
The sample after complete resection

**Fig. 8:**
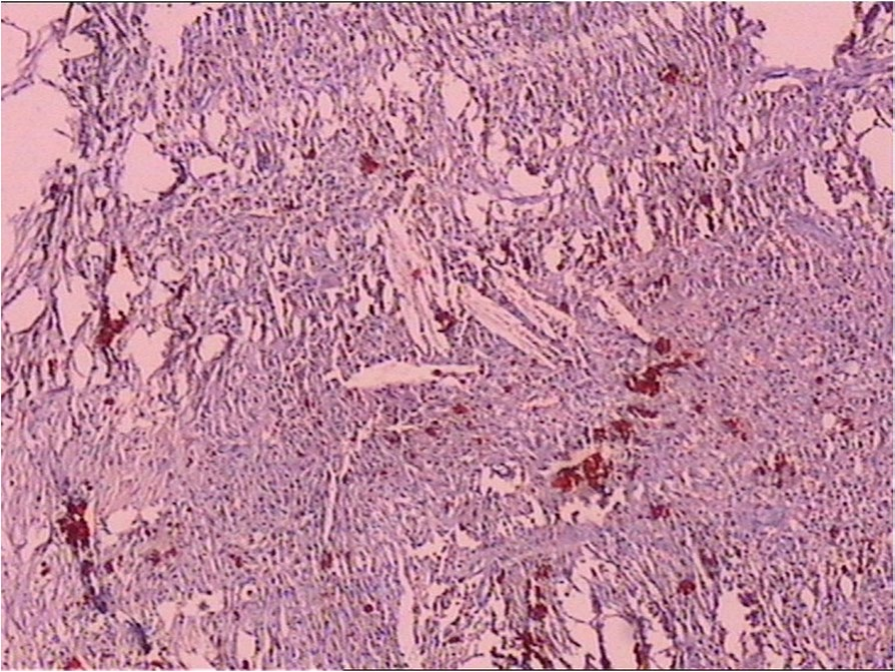
The histopathological examination of the cyst contents and wall revealed cholesterol clefts, multinucleated giant cells, histiocytes, foamy macrophages, fiber textures, altered blood pigments, and calcifications, which was consistent with the diagnosis of cholesterol granuloma

A 27-year-old East Asian male patient presented with proptosis and eyelid swelling in the left eye for ten days. There was no associated pain and history of trauma. The levels of serum low density lipoprotein cholesterol and triglyceride were higher, and apolipoprotein A1 was lower than normal. Other blood and urine tests were normal. On ocular examination, the best-corrected visual acuity was 20/20 in the right eye and 20/33 in the left eye. The patient presented with proptosis, inwards and downward eyeball displacement, and upward restricted extraocular muscle movements. Hertel exophthalmometry readings were 15 mm in the right eye and 17 mm in the left eye. An irregularly shaped soft lesion was palpable in the superior space of left orbit. The rest of ocular examination was normal. CT scan exhibited an irregularly shaped promiscuous dense occupying lesion in the left frontal bone, associated with bony erosion (Fig. 5). This lesion resulted in the bone absorption in the frontal bone and orbital roof, which penetrated down into the orbit. The boundary was relatively clear, and speckled high-density shadows could be seen inside. MRI displayed an irregular mass, the lesions were hyperintense signals on T1WI, and the lesions were isointense and hyperintense mixed signals with a thick hypointense ring around the lesions on T2WI. Contrast-enhanced imaging revealed that most parts of the tumor display a non-significant enhancement (Fig. 6). Resection was performed via an anterior orbitotomy. A dark brown mass was seen intraoperatively (Fig. 7). Management was by total excision of the lesion with curettage of the eroded bone to prevent a recurrence. The histopathological examination of the cyst contents and wall revealed cholesterol clefts, multinucleated giant cells, histiocytes, foamy macrophages, fiber textures, altered blood pigments, and calcifications, which was consistent with the diagnosis of cholesterol granuloma (Fig. 8). There was no recurrence upon three-year follow-up.

## Discussion and conclusions

Orbital cholesterol granuloma is a relatively rare condition caused by a foreign body reaction against cholesterol crystals. It predominantly occurs in young or middle-aged men [1]. The orbitofrontal cholesterol granuloma was initially described in 1902 by Denig [2, 3]. Other terms such as lipid granuloma, cholesteatoma, chronic haematic cyst, xanthomatosis, and histiocytic granuloma are also used for orbital cholesterol granuloma. However, these terms are inaccurate, and cholesterol granuloma is the most appropriate [1]. However, histopathology confirmed that the mass contained cholesterol crystals, fibrous envelope, hemosiderin, granuloma, and other components, but the pathogenesis of cholesterol granuloma is still unclear. In the petrous, the pathogenesis is believed to be caused by ventilatory obstruction of the pneumatized temporal bone resulting in local tissue breakdown and deposition of cholesterol which then crystallizes and stimulates a granulomatous reaction [5]. However, this mechanism does not apply to orbital cholesterol granuloma. In orbit, orbital hemorrhage of any cause, such as minor trauma, or spontaneous bleeding in an anticoagulant-treated patient, is the initiating event for cholesterol granuloma formation [5]. Blood-derived products degrade cholesterol and deposit crystals that stimulate the growth of surrounding tissue to form granulomas [6, 7]. Nevertheless, only a minority of cases have a history of trauma [8]. Moreover, neither of the two patients reported in this study had a history of trauma. It was speculated that a very mild trauma may have occurred inadvertently. Spontaneous bleeding caused by abnormal coagulation or blood vessel development could be one of the possibilities [9].

Cholesterol granuloma may arise within the diploe of frontal bone [10], enlarge and erode through the outer table of the bone to extraperiosteal space above the lacrimal fossa. The lesion causes ophthalmological symptoms of upper eyelid swelling, ptosis, ocular distention, proptosis, hypoglobus, extraocular movement restriction, and diplopia. The typical features observed on CT are a well-defined lesion in the superolateral orbit, isodense with the brain, and extensive erosion adjacent to the frontal bone and lacrimal fossa. The typical findings on contrast-enhanced imaging are a non-enhancing mass that presented predominantly hyperintense and heterogeneous on both T1WI and T2WI, whereas the hyperintense signal is characteristic of chronic bleeding. The paramagnetic effect of haem iron (Fe^3+^) in methemoglobin results in the characteristic high signal on all images reflecting a shortening of the T1WI involved tissue and a lengthening of the T2WI [1].

Orbitofrontal cholesterol granuloma is rare, has a prediliction to the frontal sinuses. The location of disease is similar to the frontal sinus mucoceles. Therefore, the two patients were misdiagnosed before surgery and should be differentiated from them. Mucoceles are expansive cysts of mucus secreted by goblet cells interspersed in the ciliated mucosa [11]. Although all paranasal sinuses are susceptible, the frontal and ethmoidal sinuses have the highest incidence [11]. They can occur at any age, but most patients with mucoceles are between 40- 60 [12–14]^.^ Compromised sinus ventilation is the basic etiology of mucocele formation [13, 15]. The reasons may include (1) lesions of the nasal cavity and sinuses (or abnormal development), such as nasal polyps, sinus osteoma, and nasal septum deviation, and (2) inflammation or trauma leading to nasal adhesion and sinus obstruction [16]. Mucoceles can compress and erode the surrounding bone wall, leading to absorption of the orbital wall as they grow gradually [11, 17]. Frontal sinus mucoceles frequently lead to the absorption of superior orbital wall, extending into the orbit, resulting in ocular symptoms and signs. The ocular manifestations are similar to orbital cholesterol granuloma. CT scan shows expansive growth of the mucoceles in the sinuses with absorption and destruction of adjacent bone and intrusion into surrounding structures. The clear boundary; the internal density is more uniform, mostly low density; a few can be equal or even high density. The density is dependent on the protein concentration of sac fluid. When protein concentration is high, density is high, whereas when protein concentration is low, density is low. Mucocele MRI signal intensity is quite distinct, and signal level may correlate with disease duration. Subsequently, patients with a short disease course have more moisture and hypointense presentation on T1WI. Patient with a long period of the disease has more protein, presenting hyperintense on T1WI [18].

The mainstay of treatment for cholesterol granuloma is surgical resection with curettage of the abnormal bone to reduce the recurrence of disease. Cholesterol granuloma has typical histopathological features with cholesterol clefts, multinucleated giant cells, histiocytes, foamy macrophages, fiber textures, altered blood pigments, and calcifications.

In conclusion, orbital cholesterol granuloma is relatively rare, has a prediliction to the frontal sinuses. Suppose the orbital CT shows a similar cystic lesion with irregular bone destruction in the superolateral orbit. In that case, MRI displays that the lesions are hyperintense signals on T1WI and T2WI, and the contrast-enhanced imaging reveals that most of the tumors are not significantly enhanced. The diagnosis of orbitofrontal cholesterol granuloma should be considered.

## Data Availability

The data and materials used and/or analyzed during the present study are available from the corresponding author on reasonable request.

## Abbreviations

CT: Computed tomography
MRI: Magnetic resonance imaging
T1WI: T1 weighted images
T2WI: T2 weighted images

## References

[1] Chow LP, McNab AA (2005). Orbitofrontal cholesterol granuloma. J Clin Neurosci.

[2] Hughes JD, Jacob JT, Garrity JA, Salomao DR, Link MJ (2016). Orbitofrontal Cholesterol Granuloma: Four Case Reports and a Systematic Review of the English Literature. World Neurosurg.

[3] González-García R, Román-Romero L (2010). Cholesterol granuloma of the orbit. Otolaryngol Head Neck Surg.

[4] Yan J, Cai Y, Liu R, Lin J, Li J (2015). Cholesterol granuloma of the orbit. J Craniofac Surg.

[5] Selva D, Phipps SE, O’Connell JX, White VA, Rootman J (2003). Pathogenesis of orbital cholesterol granuloma. Clin Exp Ophthalmol.

[6] Sia DI, Davis G, Selva D (2012). Recurrent orbitofrontal cholesterol granuloma: a case report. Orbit.

[7] Rong AJ, Erickson BP, Blessing NW, Dubovy SR, Lee BW (2019). Orbital cholesterol granuloma: A report and discussion of orbital findings. Am J Ophthalmol Case Rep..

[8] Li G, Zhang C, Sun Y, Mu Q, Huang H (2018). Xanthogranulomatous pituitary adenoma: A case report and literature review. Mol Clin Oncol.

[9] Shrirao N, Mukherjee B, Krishnakumar S, Biswas J (2016). Cholesterol granuloma: a case series & review of literature. Graefes Arch Clin Exp Ophthalmol.

[10] Hsu HT, Liao WC, Wu CC, Lai PH (2017). Cholesterol granuloma of the orbit. Kaohsiung J Med Sci.

[11] Scangas GA, Gudis DA, Kennedy DW (2013). The natural history and clinical characteristics of paranasal sinus mucoceles: a clinical review. Int Forum Allergy Rhinol.

[12] Courson AM, Stankiewicz JA, Lal D (2014). Contemporary management of frontal sinus mucoceles: a meta-analysis. Laryngoscope.

[13] Obeso S, Llorente JL, Pablo Rodrigo J, Sánchez R, Mancebo G, Suárez C (2009). Mucoceles de senos paranasales. Nuestra experiencia en 72 pacientes [Paranasal sinuses mucoceles. Our experience in 72 patients]. Acta Otorrinolaringol Esp..

[14] Chiu AG, Vaughan WC (2004). Management of the lateral frontal sinus lesion and the supraorbital cell mucocele. Am J Rhinol.

[15] Bosmans F, Vanhoenacker F (2020). Giant Frontal Paranasal Mucocele: Case Report and Review of the Literature. J Belg Soc Radiol..

[16] Pinarci M, Haas C, Flaig MJ, Oppel EM (2020). Frontale Mukozele [Frontal sinus mucocele]. Hautarzt.

[17] Tan CS, Yong VK, Yip LW, Amrith S (2005). An unusual presentation of a giant frontal sinus mucocele manifesting with a subcutaneous forehead mass. Ann Acad Med Singap.

[18] Bouatay R, Aouf L, Hmida B (2019). The role of imaging in the management of sinonasal mucoceles. Pan Afr Med J..

